# Alcohol Consumption and Risk of Glioma: A Meta-Analysis of 19 Observational Studies

**DOI:** 10.3390/nu6020504

**Published:** 2014-01-27

**Authors:** Zhen-Yu Qi, Chuan Shao, Chao Yang, Zhong Wang, Guo-Zhen Hui

**Affiliations:** Department of Neurosurgery, The First Affiliated Hospital of Soochow University, Suzhou 215006, China; E-Mails: qizhenyu@suda.edu.cn (Z.-Y.Q.); 20114232107@suda.edu.cn (C.Y.); wzsuda@163.com (Z.W.); hgzsuda@163.com (G.-Z.H.)

**Keywords:** glioma, alcohol drinking, ethanol, meta-analysis, risk factor

## Abstract

The relationship between risk of glioma and alcohol consumption has been widely studied, but results have been conflicting. We therefore conducted a meta-analysis of observational studies to systematically assess the relationship between alcohol drinking and risk of glioma. Two electronic databases (PubMed and EMBASE) were searched from inception to 8 August 2013 to identify pertinent studies that linked alcohol drinking with glioma risk. We used a random-effects model to calculate the overall relative risk (RR) with corresponding 95% confidence intervals (CIs). Fifteen case-control and four cohort studies were identified for this analysis. The combined RR for total alcohol drinkers *versus* non-drinkers was 0.96 (95% CI: 0.89–1.04). In the subgroup analysis by geographic area, a significant association was observed in North American studies (RR = 0.78, 95% CI: 0.65–0.93), but not in European or Asian/Australian studies. In the subgroup analysis by study design, a borderline significant association emerged in population-based case-control studies (RR = 0.82, 95% CI: 0.68–0.99), but not in hospital-based case-control studies (RR = 1.00, 95% CI: 0.99–1.01) or cohort group (RR = 1.03, 95% CI: 0.88–1.20). Our results show no material association between alcohol consumption and risk of glioma existed. Further prospective evidences are needed to confirm this association.

## 1. Introduction

The cause of glioma, the most frequent type of primary malignant brain tumor, remains largely unknown [1]. With the exception of genetic syndromes, ionizing radiation is the only well-established risk factor for glioma [1]. Indeed, these unusual exposures could explain only a minority of exposed individuals, indicating that other factors like dietary, occupational exposures and other personal and residential exposures may play a critical role in the carcinogenesis of glioma [2].

It is widely accepted that intake of alcoholic beverages is one of the most important lifestyle-related risk factor for human cancer after tobacco smoking [3]. It has been estimated that in 2002, 3.6% of all cancers (5.2% in men, 1.7% in women) are attributable to alcohol consumption worldwide [3,4]. A causal association has been confirmed between alcohol consumption and cancers of the oral cavity, pharynx, larynx, oesophagus, liver, colon, rectum, and female breast [5]. However, whether a causal link between alcohol consumption and risk of glioma exists is still unclear. To clarify this issue, we therefore conducted a meta-analysis of published observational studies.

## 2. Material and Methods

### 2.1. Search Strategy

Two authors (CS and ZYQ) independently conducted a systematic literature search of the PubMed and EMBASE databases for relevant reports published from database inception through 8 August 2013. Our research strategy was based on the following terms: (“glioma” OR “astrocytoma” OR “glioblastoma” OR “oligodendroglioma” OR “oligoastrocytoma” OR “brain cancer” OR “brain neoplasm” OR “brain tumor”) AND (“alcohol” OR “ethanol” OR “alcoholic beverages” OR “alcohol drinking” OR “beer” OR “spirits” OR “wine” OR “liquor”). No language limitation was imposed. In additional, a manual search of relevant studies’ references was performed for additional studies.

### 2.2. Inclusion Criteria

We adopted the following inclusion criteria: (1) a study of any alcohol consumption and glioma; (2) case-control or cohort design; (3) estimates of relative risk (odd ratio (OR), hazard ratio (HR), rate ratio) with corresponding 95% CIs were provided (or enough data to calculate them); (4) when more than one report based on the same study population was published, only the most comprehensive publication was included in this meta-analysis; (5) we excluded the studies which concerned total brain tumors or central nervous system (CNS) tumors in their subjects because total brain tumors or CNS tumors contain other types of tumors (*i.e.*, meningioma, pituitary adenomas, neurilemmomas), which differ from glioma in a pathological and clinical point of view; and (6) we also precluded the studies which involved childhood glioma in relation to consumption of any alcohol by the mother.

### 2.3. Data Extraction and Assessment of Methodological Quality

The data extraction and evaluation of study quality were conducted independently by two reviewers (CS and ZYQ). The following data were extracted: first author’s last name, year of publication, country, study period/follow-up years, study design, sample size, criteria for diagnosis of glioma, proxy interview (yes/no), exposure variables (beer, wine, spirits, and/or total alcohol), method of assessment of exposure (in-person interview, phone interview, and self-administered questionnaire), matching factors and covariates for adjustments, and the risk estimates with corresponding 95% CIs. The quality of included studies was assessed by using the Newcastle-Ottawa Scale (NOS) [6]. The NOS yields results from zero to nine stars. When a study gets more than six stars, it would be considered to be of relatively higher quality; otherwise it is deemed to have relatively lower quality. Any discrepancies were resolved by discussion.

### 2.4. Statistical Analysis

All statistical analyses were carried out with the STATA software [7]. The RR was used as the measure of association between alcohol drinking and glioma risk. Since the prevalence of glioma was relatively low, ORs and HRs were directly considered as RRs [8]. The study-specific adjusted RRs were extracted for meta-analysis; however, when unavailable, the raw data were used. The Random-effects model, which incorporates both within and between-study heterogeneity, was adopted to pool the risk estimates [9]. Statistical heterogeneity across studies was evaluated by the Q statistic and *I*^2^ statistic [10,11]. For the Q statistic, *p* > 0.1 was considered statistically insignificant [10]. Subgroup analyses were performed according to study design, geographic area, adjustment status, study quality, and type of alcohol consumption. Sensitivity analysis was carried out as previously described [12]. Briefly, to assess the influence of an individual study to the overall results, one study each turn was excluded from the sensitivity analysis. We also conducted a publication bias analysis through Egger’s test [13] or Begg’s funnel plot [14]. For the Egger’s test, *p* < 0.05 was considered statistically significant.

As the most common definition of alcohol consumption is ever intake of alcohol, our main analysis is “ever any alcohol drinkers *versus* nondrinkers”. One study which used non-regular drinkers as reference was also included in this meta-analysis [15]. Alcohol intake was measured by different units. Therefore, we converted alcohol consumption categories into grams of ethanol per day as a standard measure of alcohol intake (assuming one drink = 12.5 g, 1 mL = 0.8 g, 1 oz = 28.35 g of ethanol) [16]. The levels of alcohol intake were reported by a range, we assigned to each class the dose corresponding to the midpoint of the range (for the open-ended upper category, the exposure alcohol doses were calculated as 1.2 times the lower bound) [16]. Moderate alcohol drinking was defined as consumption of <25 g/day of ethanol, and heavy as consumption of ≥25 g/day of ethanol [16]. When in a particular study more than one category fell in the exposure level considered, we calculated a combined risk estimates with Hamling’s method [17]. This method was used for pooled estimates using the same reference category or the same set of controls, taking into account association between estimates.

## 3. Results

### 3.1. Search Results and Description of Studies

**Figure 1:**
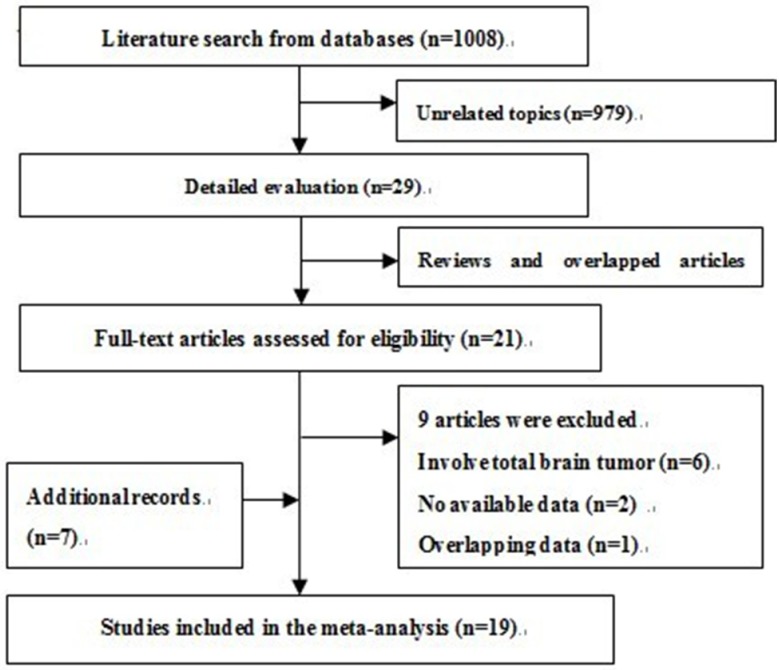
Flow diagram of study selection.

Figure 1 presents the flowchart of study selection process. The literature searches yielded a total of 1008 articles: 416 from PubMed and 592 from EMBASE. After careful review, 21 studies were identified for full-text assessment. Nine of 21 studies were further excluded for the following reasons: involving total brain tumors or CNS tumors [18,19,20,21,22,23], no available data [24,25], and overlapping data [26]. Seven additional studies were identified from the reference lists of relevant studies. Therefore, 19 studies (fifteen case-control and four cohort studies) included data suitable for our meta-analysis [15,27,28,29,30,31,32,33,34,35,36,37,38,39,40,41,42,43,44]. Studies were published from 1970 to 2011. Of 19 studies, seven originated from USA [27,31,32,33,37,39,40], three from Australia [34,36,43], two from Italy [28,35], one from Sweden [29], one from Canada [30], one from China [38], one from the UK [41], one from Greece [42], one from France [44], and one from the USA, Sweden and Denmark [15]. The vast majority of cases were histologically identified, while case definition was based on radiological criteria for some cases. Of 19 studies, twelve provided data on total alcohol intake [15,27,29,32,34,36,39,40,41,42,43,44]; nine on beer [30,31,33,34,36,37,38,39,43]; eight on wine [28,30,31,34,35,36,39,43], and six on spirits [30,31,34,36,38,39]. Controls were recruited randomly from hospitals or the general population. Data for drinking habits were ascertained by phone interview, in person interview, or self-administered questionnaire. More details of included studies were shown in Table 1. As shown in Table 1, thirteen studies were awarded six or more stars [29,31,32,33,34,35,36,37,38,39,40,41,43], indicating that the overall quality of the studies was relatively higher. Thus, the remaining six studies were considered to have relatively lower quality [15,27,28,30,42,44].

**Table 1:** Characteristics of studies included in the meta-analysis.

Study	Country	Study Design	Sample Size	Case Diagnosis	Exposure Variables	Exposure Assessment ^†^	Matching or Adjustments ^‡^	Study Quality
Choi, 1970 [27]	USA	HCC	55/55	Medical records	Total alcohol	a	1,2,3,4,14	5
Musicco, 1982 [28]	Italy	HCC	47/196	Medical records	Wine	a	1,2,4	5
Ahlbom, 1986 [29]	Sweden	PCC	78/92	Medical records	Total alcohol	c	1,2,4	6
Burch, 1987 [30]	Canada	HCC	215/215	Pathology reports	Beer, wine, spirits	a	1,2,4,9,20	5
Preston-Martin, 1989 [31]	USA	PCC	202/202	Pathology reports	Beer, wine, spirits	a	1,2,4	6
Mills, 1989 [32]	USA	Cohort	21/34,000	Cancer registry	Total alcohol	c	1,2	6
Hochberg, 1990 [33]	USA	PCC	160/128	Medical records	Beer	b, c	1,2,4,10	7
Ryan, 1992 [34]	Australia	PCC	110/417	Medical records	Total alcohol (beer, wine, spirits)	a, c	1,2,4	6
Zampieri, 1994 [35]	Italy	HCC	195/195	Pathology reports	Wine	a	1,2,4,14,20	6
Hurley, 1996 [36]	Australia	PCC	416/420	Pathology reports	Total alcohol (beer, wine, spirits)	a	1,2,20	6
Blowers, 1997 [37]	USA	PCC	94/94	Cancer registry	Beer	a	1,3	6
Hu, 1998 [38]	China	HCC	218/436	Pathology reports	Beer, spirits	a	1,2,4,5,9,15,16	6
Efird, 2004 [39]	USA	Cohort	130/133,811	Cancer registry	Total alcohol (beer, wine, spirits)	c	1,2,3,5,6,8	7
Ruder, 2006 [40]	USA	PCC	798/1175	Pathology reports	Total alcohol	a, b	1,2,4,5	7
Benson, 2008 [41]	UK	Cohort	646/1,249,670	Cancer registry	Total alcohol	c	1,4,6,10,11,12, 13,14,17,18, 19	6
Gousias, 2009 [42]	Greece	HCC	56/82	Medical records	Total alcohol	a	1,2,4	5
Baglietto, 2011 [43]	Australia	Cohort	67/39,766	Cancer registry	Total alcohol (beer, wine)	a	2,4,5,8,16	8
Cabaniols, 2011 [44]	France	HCC	122/122	Pathology reports	Total alcohol	a, c	1,2	5
McCarthy, 2011 [15]	USA, Sweden, Denmark	PCC/HCC	617/1260	Pathology reports	Total alcohol	a, b, c	1,2,3,4,20	5

PCC, population-based case-control study; HCC, hospital-based case-control study; ^†^ Assessment tools to get information of alcohol drinking consisted of: (a) in-person interview, (b) phone interview, (c) self-administered questionnaire; ^‡^ Matching or adjustments were: (1) age, (2) sex, (3) race, (4) area of residence, (5) education, (6) smoking, (7) alcohol/beer/spirit, (8) coffee, (9) income/marital status, (10) socioeconomic status, (11) exercise, (12) height, (13) body mass index, (14) hospital of admission, (15) occupational exposure, (16) consumption of vegetables and fruit, (17) parity, (18) age at first birth, (19) oral contraception, (20) interview year for control/date of diagnosis for cases.

### 3.2. Overall Association of Alcohol Consumption and Risk of Glioma

**Figure 2:**
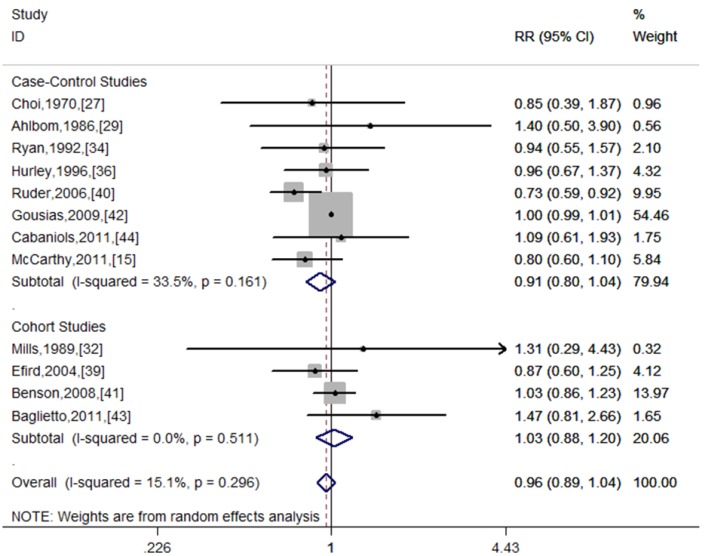
Forest plot for total alcohol drinkers *versus* non-drinkers.

Twelve studies evaluated the association between alcohol consumption and risk of glioma [15,27,29,32,34,36,39,40,41,42,43,44]. Figure 2 presents the forest plots for total alcohol drinkers *versus* non-drinkers. The pooled RR was 0.96 (95%: 0.89–1.04, *I*^2^ = 15.1%, *p* for heterogeneity = 0.296).

### 3.3. Stratifying Analysis

Stratifying by study design: the combined RR was 1.03 (95% CI: 0.88–1.20, *I*^2^ = 0.0%, *p* for heterogeneity = 0.511) for cohort studies. For case-control studies, the combined RR was 0.91 (95% CI: 0.80–1.04, *I*^2^ = 33.5%, *p* for heterogeneity = 0.161). In further analysis according to the source of control, no significant association was observed for hospital-based case-control studies (RR = 1.00, 95% CI: 0.99–1.01, *I*^2^ = 0.0%, *p* for heterogeneity = 0.882), while significant association was detected for population-based case-control studies (RR = 0.82, 95% CI: 0.68–0.99, *I*^2^ = 3.2%, *p* for heterogeneity = 0.377).

Stratification by geographic area: the pooled RRs were 1.00 (95% CI: 0.99–1.01, *I*^2^ = 0.0%, *p* for heterogeneity = 0.896), 0.78 (95% CI: 0.65–0.93, *I*^2^ = 0.0%, *p* for heterogeneity = 0.773), and 1.04 (95% CI: 0.80–1.35, *I*^2^ = 0.0%, *p* for heterogeneity = 0.441) for Europe, North America, and Asia/Australia, respectively.

Stratification by adjustment status: no significant link was found in unadjusted studies (RR = 1.00, 95% CI: 0.99–1.01, *I*^2^ = 0.0%, *p* for heterogeneity = 0.947), whereas a borderline significant association in adjusted groups (RR = 0.86, 95% CI: 0.74–1.00, *I*^2^ = 11.5%, *p* for heterogeneity = 0.342). In subgroup analysis by study quality, no significant correlation was observed in the high quality group (Table 2). For the low quality group, a similar trend was detected (Table 2).

**Table 2:** Results of meta-analysis for alcohol consumption and risk of glioma.

Group	Number of Studies	Summary Effect		Heterogeneity	
		RR (95% CI)	*p* Value	*I*^2^	*p*
All studies	12	0.96 (0.89–1.04)	0.312	15.1%	0.296
**Study design**					
Case-control	8	0.91 (0.80–1.04)	0.164	33.5%	0.161
PB	4	0.82 (0.68–0.99)	0.034	3.2%	0.377
HB	3	1.00 (0.99–1.01)	1.000	0.0%	0.882
Cohort	4	1.03 (0.88–1.20)	0.734	0.0%	0.511
**Geographic area**					
Europe	4	1.00 (0.99–1.01)	0.977	0.0%	0.896
North America	4	0.78 (0.65–0.93)	0.007	0.0%	0.773
Asia/Australia	3	1.04 (0.80–1.35)	0.777	0.0%	0.441
**Adjustment status**					
Adjusted	7	0.86 (0.74–1.00)	0.058	11.5%	0.342
Unadjusted	5	1.00 (0.99–1.01)	0.988	0.0%	0.947
**Type of alcohol**					
Beer	9	0.95 (0.81–1.10)	0.484	0.0%	0.443
Wine	8	0.92 (0.71–1.20)	0.548	64.4%	0.006
Spirits	6	1.17 (0.98–1.41)	0.09	5.3%	0.383
**Study quality**					
High	8	0.94 (0.81–1.09)	0.383	23.1%	0.245
Low	4	1.00 (0.99–1.01)	0.962	0.0%	0.507

**Figure 3:**
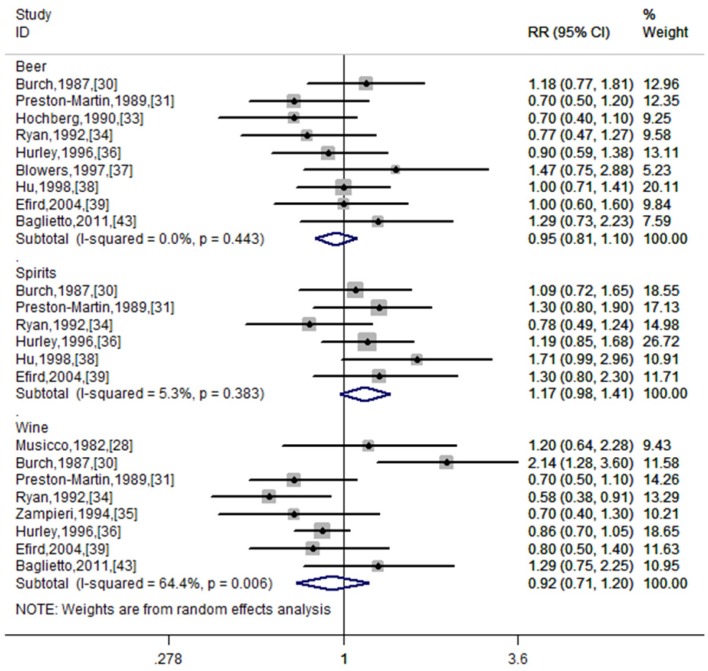
Forest plot for the forest plots for special-types of alcohol drinkers *versus* non-drinkers.

Stratification by type of alcohol consumption: the pooled RRs for beer, wine, and spirits were 0.95 (95% CI: 0.81–1.10, *I*^2^ = 0.0%, *p* for heterogeneity = 0.443), 0.92 (95% CI: 0.71–1.20, *I*^2^ = 64.4%, *p* for heterogeneity = 0.006), and 1.17 (95% CI: 0.98–1.41, *I*^2^ = 5.3%, *p* for heterogeneity = 0.383), respectively. Figure 3 shows the forest plots for special-types of alcohol drinkers *versus* non-drinkers.

### 3.4. Sensitivity Analysis and Publication Bias

**Figure 4:**
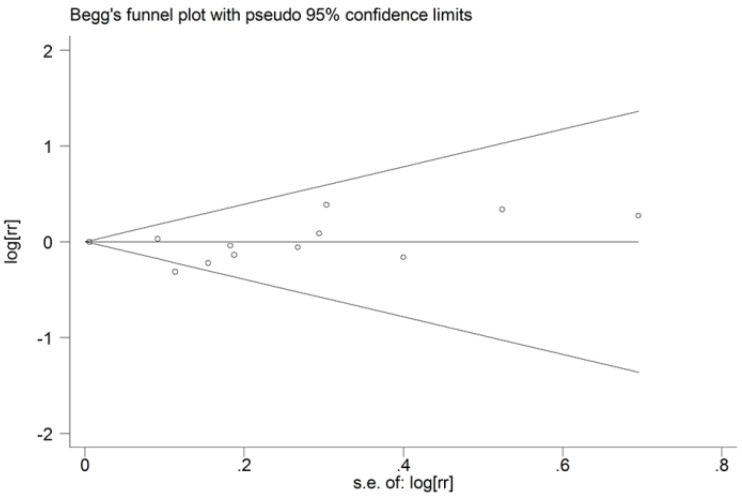
Funnel plot analysis to detect publication bias.

We performed a sensitivity analysis to evaluate the influence of individual study on the overall results by omitting one study each turn. The results of sensitivity analysis were not meaningfully altered (data not shown). The Begg’s funnel plot does not show any asymmetry (Figure 4), indicating that no evidence of publication bias were detected. Also, the Egger’s test suggested there is no publication bias (*p* for Egger’s test = 0.465).

## 4. Discussion

The association between alcohol consumption and risk of glioma has long been explored with conflicting results. The studies involved the questions about total alcohol consumption and a specific-type of alcoholic beverage. One previous meta-analysis of 19 studies has been conducted to quantitatively assesse the relationship between alcohol drinking and brain tumor [16]. Galeone and colleagues found that alcohol consumption did not appear to be associated with brain cancer. In a dose-response analysis, a moderate increase in risk of brain tumor for intakes of two or more drinks per day [16]. It is well known that brain tumors are a heterogeneous group of tumors which vary in tissue origins, invasive potential and prognosis. Thus, an analysis of combination of glioma and meningioma may be the result of some unknown bias and make these findings more confounding. In order to derive a more accurate estimation of the association between alcohol intake and glioma, an updated meta-analysis was performed. In our meta-analysis, those studies involving total brain tumors or meningioma were excluded [17,18,19,20,21,22,45,46,47]. Thus, a final total of 19 studies were identified for our analysis. Our analyses indicated that the risk of glioma did not appear associated with alcohol intake.

In subgroup analysis by geographic area, a significant association was observed in North American studies, but this correlation did not emerge for European or Asian/Australian studies. When subgroup analyses were performed according to study design, no significant associations were observed in cohort or hospital-based case-control studies. However, in population-based case-control studies, an 18% decreased risk was detected among alcohol drinkers compared with nondrinkers. The significance of these findings is unclear. Therefore, additional studies are warranted to confirm these findings.

We also evaluated the correlation between risk of glioma and specific-types of alcoholic beverage. No associations emerged with beer, wine, or spirits. These findings were consistent with all studies except two [30,34]. In a hospital-based case-control study, Burch and colleagues found that wine consumption was associated with an elevated risk of glioma (RR = 2.14, 95% CI = 1.28–3.60) [30]. However, a lower risk (RR = 0.58, 95% CI = 0.38–0.91) was observed in a population-based case-control study with 110 cases and 417 controls [34]. The two risk estimates were not adjusted for any confounders. Thus, residual confounding was possible. Furthermore, two studies have investigated several types of wine in relation to glioma [34,36]. The study conducted by Ryan found a decreased risk for drinkers of white wine (RR = 0.53, 95% CI = 0.33–0.85) and red wine (RR = 0.33, 95% CI = 0.33–1.08), although this correlation for red wine was not statistically significant [34]. In the latter study of Hurley, no meaningful associations were observed for white, red or fortified wine [36].

A dose-response relationship in a meta-analysis supports to a suspected causal relationship between exposure and disease. Three studies have examined the relationship between risk of glioma and different levels of alcohol consumption [36,39,43]. Two studies shown non-significant increase or decrease in risk emerged [36,39]. However, in the Melbourne Collaborative cohort study, Baglietto and colleagues found an 16% increase risk for each additional 10 g/day and people drinking 40 g/day of alcohol or more had up to three-fold higher risk relative to nondrinkers [43]. The discrepancy may be due to the limitations of statistical power and different levels of exposure defined in each study. To overcome these limitations, universal standards (moderate alcohol drinking was defined as consumption of <25 g/day of ethanol, and heavy as consumption of ≥25 g/day of ethanol) were adopted and the dose-risk analysis was performed. Our results still showed no significant association between risk of glioma and moderate or heavy intake of alcohol. However, significant heterogeneity was found (*I*^2^ = 63.3%). Moreover, only three studies were identified for dose-response analysis. Therefore, the relationship between glioma and alcohol drinking needs further discussion.

Although we found that alcohol consumption was not asssociated with glioma risk, various mechanisms have been proposed. First, alcohol is capable of traversing the blood-brain barrier and has been considered as an established factor for several other tumors or diseases [16]. Therefore, alcohol could play a carcinogenic role in the brain directly. Second, acetaldehyde and reactive oxygen species, the products of alcohol metabolism, are toxic to cells when they react with proteins, lipids, and DNA [43]. Moreover, acetaldehyde has been demonstrated to be a neurocarcinogen in animals [43]. Finally, alcohol contains *N*-nitroso compounds, which result in brain tumors in animals [36,43].

Of note, several limitations should be addressed in our analysis. First, since our meta-analysis was based on observational studies, confounding factors are often of concern. As we performed an analysis limited to those studies that provided adjusted risk estimates, a 14% decreased risk of glioma was observed among alcohol drinkers. Thus, we cannot rule out the probability that our findings were due to confounding from other risk factors. Second, involving specific-type of alcohol drinking could result in an underestimation of the risk associated with the true amount of alcohol consumed. Third, we were unable to assess separately various types of glioma (e.g., astrocytoma, oligodendroglioma, glioblastoma, *etc*.) because limited data were eligible. Fourth, a separate analysis for females and males was not possible since only two studies provided results separately for men and women. Epidemiological data have shown the incidence of glioma is 1.5–2 fold higher in men than in women [12,48]. Fifth, most evidence was retrospective. Thus, the possible recall and selection bias may confound the relationship. Finally, potential publication bias may distort the association between alcohol consumption and risk of glioma.

## 5. Conclusions

This meta-analysis provides evidence of a lack of association between alcohol consumption and risk of glioma.
